# 5-(Hy­droxy­meth­yl)furan-2-carb­oxy­lic acid

**DOI:** 10.1107/S1600536811019489

**Published:** 2011-05-28

**Authors:** Jun-Liang Liu, Qing-Yan Xu

**Affiliations:** a Laboratory of Microbial Pharmaceutical Engineering, Xiamen University, Xiamen 361005, People’s Republic of China

## Abstract

In the title compound, C_6_H_6_O_4_, the furan ring is nearly coplanar with the carboxyl group, the maximum atomic deviation being 0.0248 (9) Å. The crystal packing is stabilized by classical O—H⋯O and weak C—H⋯O hydrogen bonding.

## Related literature

For the biochemical significance of the title compound, see: Mrochek & Rainey (1972 ▶).
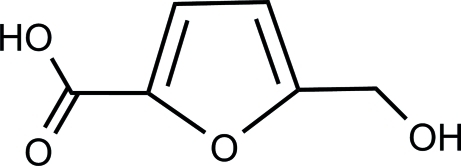


## Experimental

### 

#### Crystal data

C_6_H_6_O_4_
*M* *_r_* = 142.11
Orthorhombic, 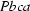

*a* = 10.838 (3) Å
*b* = 7.2601 (17) Å
*c* = 15.526 (4) Å
*V* = 1221.7 (5) Å^3^
*Z* = 8
Mo *K*α radiation
μ = 0.13 mm^−1^
*T* = 294 K
0.32 × 0.22 × 0.12 mm

#### Data collection

Bruker SMART CCD area-detector diffractometer
5637 measured reflections
1098 independent reflections
1033 reflections with *I* > 2σ(*I*)
*R*_int_ = 0.025

#### Refinement

*R*[*F* ^2^ > 2σ(*F* ^2^)] = 0.035
*wR*(*F* ^2^) = 0.090
*S* = 1.08
1098 reflections
99 parameters
H atoms treated by a mixture of independent and constrained refinement
Δρ_max_ = 0.19 e Å^−3^
Δρ_min_ = −0.21 e Å^−3^

### 

Data collection: *SMART* (Bruker, 2001 ▶); cell refinement: *SAINT* (Bruker, 2001 ▶); data reduction: *SAINT*; program(s) used to solve structure: *SHELXS97* (Sheldrick, 2008 ▶); program(s) used to refine structure: *SHELXL97* (Sheldrick, 2008 ▶); molecular graphics: *ORTEP-3* (Farrugia, 1997 ▶); software used to prepare material for publication: *SHELXL97*.

## Supplementary Material

Crystal structure: contains datablocks I, global. DOI: 10.1107/S1600536811019489/xu5218sup1.cif

Structure factors: contains datablocks I. DOI: 10.1107/S1600536811019489/xu5218Isup2.hkl

Supplementary material file. DOI: 10.1107/S1600536811019489/xu5218Isup3.cml

Additional supplementary materials:  crystallographic information; 3D view; checkCIF report

## Figures and Tables

**Table 1:** Hydrogen-bond geometry (Å, °)

*D*—H⋯*A*	*D*—H	H⋯*A*	*D*⋯*A*	*D*—H⋯*A*
O1—H1*A*⋯O3^i^	0.87 (2)	1.83 (2)	2.6951 (16)	169.2 (19)
O4—H4*A*⋯O1^ii^	0.96 (2)	1.61 (2)	2.5643 (17)	171 (2)
C7—H7*A*⋯O4^iii^	0.97	2.45	3.265 (2)	142

Symmetry codes: (i) 
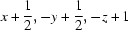
; (ii) 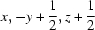; (iii) 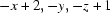.

**Fig. 1.**
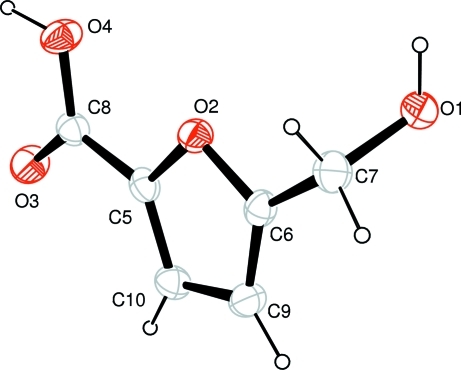
The molecular structure of the title compound with 50% probability ellipsoids.

## supplementary crystallographic information

### Comment

In the molecular structure of the compound, all the carbon atoms locate in the same plane. The crystal packing is stabilized by O—H···O and weak C—H···O hydrogen bonding (Table 1).

### Experimental

The title compound was obtained from the fermentation of *Aspergillus sp.* by column chromatography. The strain was isolated from a soil sample collected from Jinjiang salt-field, Fujian, China. The strain was cultured using half sea-water Potato Dextrose Agar medium at 28 degrees celsius for 14 days. The fermentation (40 liters) was extracted with ethyl acetate (EtOAc). The EtOAc extract (18.0 g) eluted with methanol-water (30%, 50%, 70%, 100%; V/V) to yield 12 fractions by column chromatography RP-18. Fraction 1 was further puried by Sephadex LH-20 (140 g) chromatography with methanol. Then 1–4 eluted with acetone by Sephadex LH-20 (80 g) chromatography, then the main components further puried by silica-gel column chromatography to afford the title compound (8.0 mg).

### Refinement

The carboxyl and hydroxy H atoms were located in a difference Fourier map and refined isotropically. Other H atoms were positioned geometrically and were treated as riding on their parent atoms, with C—H distances of 0.93–0.97 Å, and U_iso_(H) = 1.2U_eq_(C).

### Crystal data

**Table:**

C_6_H_6_O_4_	*F*(000) = 592
*M**_r_* = 142.11	*D*_x_ = 1.545 Mg m^−^^3^
Orthorhombic, *P**b**c**a*	Mo *K*α radiation, λ = 0.71073 Å
Hall symbol: -P 2ac 2ab	Cell parameters from 10988 reflections
*a* = 10.838 (3) Å	θ = 2.6–25.2°
*b* = 7.2601 (17) Å	µ = 0.13 mm^−^^1^
*c* = 15.526 (4) Å	*T* = 294 K
*V* = 1221.7 (5) Å^3^	Block, colourless
*Z* = 8	0.32 × 0.22 × 0.12 mm

### Data collection

**Table:**

Bruker SMART CCD area-detector diffractometer	1033 reflections with *I* > 2σ(*I*)
Radiation source: fine-focus sealed tube	*R*_int_ = 0.025
graphite	θ_max_ = 25.2°, θ_min_ = 2.6°
φ and ω scans	*h* = −12→12
5637 measured reflections	*k* = −8→8
1098 independent reflections	*l* = −18→13

### Refinement

**Table:**

Refinement on *F*^2^	Primary atom site location: structure-invariant direct methods
Least-squares matrix: full	Secondary atom site location: difference Fourier map
*R*[*F*^2^ > 2σ(*F*^2^)] = 0.035	Hydrogen site location: inferred from neighbouring sites
*wR*(*F*^2^) = 0.090	H atoms treated by a mixture of independent and constrained refinement
*S* = 1.08	*w* = 1/[σ^2^(*F*_o_^2^) + (0.0457*P*)^2^ + 0.5232*P*] where *P* = (*F*_o_^2^ + 2*F*_c_^2^)/3
1098 reflections	(Δ/σ)_max_ = 0.001
99 parameters	Δρ_max_ = 0.19 e Å^−^^3^
0 restraints	Δρ_min_ = −0.21 e Å^−^^3^

### Special details

**Table:**

Geometry. All esds (except the esd in the dihedral angle between two l.s. planes) are estimated using the full covariance matrix. The cell esds are taken into account individually in the estimation of esds in distances, angles and torsion angles; correlations between esds in cell parameters are only used when they are defined by crystal symmetry. An approximate (isotropic) treatment of cell esds is used for estimating esds involving l.s. planes.
Refinement. Refinement of *F*^2^ against ALL reflections. The weighted *R*-factor *wR* and goodness of fit *S* are based on *F*^2^, conventional *R*-factors *R* are based on *F*, with *F* set to zero for negative *F*^2^. The threshold expression of *F*^2^ > σ(*F*^2^) is used only for calculating *R*-factors(gt) *etc*. and is not relevant to the choice of reflections for refinement. *R*-factors based on *F*^2^ are statistically about twice as large as those based on *F*, and *R*- factors based on ALL data will be even larger.

### Fractional atomic coordinates and isotropic or equivalent isotropic displacement parameters (Å^2^)

**Table:**

	*x*	*y*	*z*	*U*_iso_*/*U*_eq_	
O1	0.93802 (10)	0.18266 (16)	0.31353 (7)	0.0276 (3)	
O2	0.84194 (9)	0.14583 (14)	0.49749 (6)	0.0216 (3)	
O3	0.68657 (9)	0.30625 (16)	0.68473 (7)	0.0300 (3)	
O4	0.88618 (9)	0.26469 (16)	0.65427 (7)	0.0282 (3)	
C5	0.74292 (13)	0.19201 (19)	0.54835 (9)	0.0204 (3)	
C6	0.79370 (13)	0.08250 (18)	0.42169 (8)	0.0208 (3)	
C7	0.88366 (14)	0.0247 (2)	0.35516 (9)	0.0262 (4)	
H7A	0.9481	−0.0487	0.3817	0.031*	
H7B	0.8424	−0.0510	0.3125	0.031*	
C8	0.76830 (13)	0.2598 (2)	0.63503 (9)	0.0219 (3)	
C9	0.66913 (13)	0.0865 (2)	0.42436 (9)	0.0243 (4)	
H9A	0.6155	0.0498	0.3809	0.029*	
C10	0.63590 (14)	0.1574 (2)	0.50616 (9)	0.0242 (3)	
H10A	0.5564	0.1765	0.5268	0.029*	
H4A	0.898 (2)	0.290 (3)	0.7143 (16)	0.060 (6)*	
H1A	1.018 (2)	0.179 (3)	0.3204 (13)	0.051 (6)*	

### Atomic displacement parameters (Å^2^)

**Table:**

	*U*^11^	*U*^22^	*U*^33^	*U*^12^	*U*^13^	*U*^23^
O1	0.0203 (6)	0.0392 (7)	0.0235 (6)	−0.0018 (5)	0.0007 (4)	0.0040 (5)
O2	0.0178 (5)	0.0265 (6)	0.0205 (5)	0.0012 (4)	0.0005 (4)	−0.0004 (4)
O3	0.0213 (5)	0.0445 (7)	0.0242 (6)	0.0059 (5)	0.0039 (4)	−0.0031 (5)
O4	0.0188 (5)	0.0425 (7)	0.0231 (6)	0.0034 (5)	−0.0003 (4)	−0.0070 (5)
C5	0.0189 (7)	0.0215 (7)	0.0209 (7)	0.0024 (5)	0.0044 (6)	0.0035 (5)
C6	0.0257 (8)	0.0178 (7)	0.0188 (7)	−0.0001 (6)	−0.0015 (6)	0.0010 (5)
C7	0.0273 (8)	0.0281 (8)	0.0232 (8)	0.0002 (6)	0.0018 (6)	−0.0014 (6)
C8	0.0188 (7)	0.0223 (7)	0.0246 (7)	0.0016 (6)	0.0018 (6)	0.0030 (6)
C9	0.0237 (8)	0.0251 (8)	0.0242 (8)	−0.0019 (6)	−0.0038 (6)	−0.0003 (6)
C10	0.0179 (7)	0.0282 (8)	0.0265 (8)	0.0002 (6)	0.0021 (6)	0.0022 (6)

### Geometric parameters (Å, °)

**Table:**

O1—C7	1.4421 (19)	C5—C8	1.459 (2)
O1—H1A	0.88 (2)	C6—C9	1.351 (2)
O2—C6	1.3675 (17)	C6—C7	1.481 (2)
O2—C5	1.3739 (16)	C7—H7A	0.9700
O3—C8	1.2224 (18)	C7—H7B	0.9700
O4—C8	1.3124 (18)	C9—C10	1.417 (2)
O4—H4A	0.96 (2)	C9—H9A	0.9300
C5—C10	1.355 (2)	C10—H10A	0.9300
			
C7—O1—H1A	109.0 (13)	O1—C7—H7B	109.5
C6—O2—C5	106.15 (11)	C6—C7—H7B	109.5
C8—O4—H4A	111.1 (13)	H7A—C7—H7B	108.1
C10—C5—O2	110.21 (12)	O3—C8—O4	123.66 (14)
C10—C5—C8	132.03 (13)	O3—C8—C5	122.60 (13)
O2—C5—C8	117.72 (12)	O4—C8—C5	113.74 (12)
C9—C6—O2	110.38 (12)	C6—C9—C10	106.82 (13)
C9—C6—C7	133.27 (13)	C6—C9—H9A	126.6
O2—C6—C7	116.35 (12)	C10—C9—H9A	126.6
O1—C7—C6	110.88 (12)	C5—C10—C9	106.44 (13)
O1—C7—H7A	109.5	C5—C10—H10A	126.8
C6—C7—H7A	109.5	C9—C10—H10A	126.8

### Hydrogen-bond geometry (Å, °)

**Table:**

*D*—H···*A*	*D*—H	H···*A*	*D*···*A*	*D*—H···*A*
O1—H1A···O3^i^	0.87 (2)	1.83 (2)	2.6951 (16)	169.2 (19)
O4—H4A···O1^ii^	0.96 (2)	1.61 (2)	2.5643 (17)	171 (2)
C7—H7A···O4^iii^	0.97	2.45	3.265 (2)	142

Symmetry codes: (i) *x*+1/2, −*y*+1/2, −*z*+1; (ii) *x*, −*y*+1/2, *z*+1/2; (iii) −*x*+2, −*y*, −*z*+1.
